# Case Report: Late Hypersensitivity Reaction to Hydroxyurea in a Patient With Myeloproliferative Disorder

**DOI:** 10.1155/crh/6693642

**Published:** 2025-10-16

**Authors:** Navkirat Kahlon, Sujatha Baddam, Harinderjeet Kaur, Prabhat Singh, Zaheer Qureshi, Anusha Manje Gowda

**Affiliations:** ^1^Department of Medical Oncology, Mass General Cancer Center at Wentworth-Douglass Hospital, Dover, New Hampshire, USA; ^2^Department of Internal Medicine, Huntsville Hospital, Huntsville, Alabama, USA; ^3^Department of Internal Medicine, Mercy Hospital, Springfield, Missouri, USA; ^4^Department of Nephrology, Kidney Specialists of South Texas, San Antonio, Texas, USA; ^5^Department of Internal Medicine, Frank H. Netter MD School of Medicine at Quinnipiac University, North Haven, Connecticut, USA; ^6^Department of Medical Oncology, Mary Washington Hospital, Fredericksburg, Virginia, USA

## Abstract

Hydroxyurea is a cornerstone therapy for myeloproliferative disorders such as early-stage myelofibrosis. However, rare hypersensitivity reactions can complicate its use and require careful management. This case describes a patient with early-stage myelofibrosis, with JAK2 V617F mutation, and Grade 1 reticulin fibrosis on bone marrow biopsy. He was started on hydroxyurea 500 mg daily. The patient developed delayed hypersensitivity characterized by fever, chills, and fatigue. Symptoms initially resolved with self-discontinuation, but rechallenge twice with alternate-day dosing led to rapid recurrence following each dose. Discontinuation of hydroxyurea resolved symptoms permanently, and the patient was transitioned to ruxolitinib for management of the underlying disease. This case highlights the importance of recognizing hypersensitivity reactions during hydroxyurea therapy and implementing alternative strategies to optimize patient outcomes.

## 1. Introduction

Hydroxyurea is widely used for managing myeloproliferative disorders, including early-stage myelofibrosis (MF), where it effectively controls thrombocytosis, reduces spleen size, and alleviates disease-related symptoms [1, 2]. However, its use is associated with a range of side effects that can impact treatment decisions.

The most common side effects of hydroxyurea include gastrointestinal disturbances such as nausea and vomiting, dermatological issues such as dry skin and hyperpigmentation, and general symptoms such as fatigue or mild headaches [3]. Severe toxicities, though rare, include myelosuppression (resulting in an increased risk of infections, anemia, and thrombocytopenia), secondary malignancies such as leukemia and skin cancer, and hypersensitivity reactions [3, 4]. Hypersensitivity reactions to hydroxyurea are uncommon but pose a significant clinical concern. Symptoms such as fever, flu-like malaise, and systemic inflammation demand prompt recognition and management. Though not widely documented in standard literature, these adverse effects can be life-threatening. Timely identification is crucial to prevent serious complications. Clinicians must remain vigilant for unexpected inflammatory responses, ensuring rapid intervention to mitigate potential risks.

This report discusses a delayed hypersensitivity reaction to hydroxyurea and reviews similar cases in the literature to provide insights into diagnosis and management strategies for this rare toxicity.

## 2. Case Presentation

A 65-year-old male presented with mild macrocytic anemia and thrombocytosis, leading to a diagnosis of early-stage MF. Diagnostic findings included Grade 1 reticulin fibrosis on bone marrow biopsy and a positive JAK2 V617F mutation. Hydroxyurea was initiated at 500 mg daily as a first-line therapy to manage the condition.

Approximately 4 weeks into therapy, the patient developed fever, chills, and extreme fatigue, which was initially attributed to a viral illness. He self-discontinued hydroxyurea for 1 week, during which all symptoms resolved entirely. In collaboration with his medical team, hydroxyurea was resumed at a reduced dosing schedule of 500 mg on Monday, Wednesday, and Friday, as the initial symptoms were presumed unrelated to the medication. However, progressively severe hypersensitivity reactions occurred. After the first dose, the patient developed a fever of 102.5 F along with systemic fatigue. Following the second dose, his fever escalated to 103.2°F, accompanied by worsening fatigue and chills. With each additional dose, symptoms continued to intensify, leading to clinical suspicion of a hypersensitivity reaction. Hydroxyurea was permanently discontinued after this.

Supportive care with acetaminophen and ibuprofen was used to manage fever and fatigue during symptomatic episodes. Hypersensitivity symptoms resolved permanently following drug cessation, without the need for further medical intervention. The patient was subsequently transitioned to ruxolitinib for management of the underlying MF. On this alternative regimen, his disease remained stable, and no recurrence of hypersensitivity symptoms was reported.

## 3. Discussion

Hypersensitivity reactions to hydroxyurea, while rare, require timely recognition and intervention to prevent complications [5–10]. Delayed hypersensitivity reactions, such as the one observed in this case, are typically mediated by a Type IV immune response, involving T-cell activation and cytokine release, leading to systemic inflammation [3, 6, 10]. Hydroxyurea-induced fever (> 38.5°C) has been documented in patients with polycythemia vera (PV), essential thrombocythemia (ET), and MF. A 2012 study analyzing hydroxyurea-related toxicity in 3411 patients with Philadelphia chromosome-negative myeloproliferative neoplasms (MPNs) reported 16 cases of fever. This study also highlighted the rarity of hydroxyurea-induced fever, noting that fewer than 30 cases had been reported in the literature then. Fever typically emerged after a median of 31 days of treatment and resolved spontaneously upon hydroxyurea discontinuation, reinforcing the presumptive diagnosis of drug-induced hypersensitivity [11].

The diagnosis is typically presumptive, based on the resolution of fever after hydroxyurea withdrawal and its recurrence upon rechallenge. The pathogenesis of this hypersensitivity reaction remains unclear, necessitating further investigation into the underlying mechanisms. Early reactions, such as fever and flu-like symptoms, typically emerge within the first days or weeks of therapy and may resolve swiftly upon discontinuation [5–8]. However, delayed hypersensitivity reactions, particularly those manifesting from weeks to months after hydroxyurea initiation, tend to be more severe and pose greater clinical risks, including life-threatening systemic inflammation [9, 10].

Cases such as hydroxyurea-induced pneumonitis demonstrate how late-onset toxicity can lead to progressive pulmonary inflammation and respiratory failure, necessitating systemic corticosteroid therapy for recovery [9, 10]. The 3-month onset period observed in fatal cases underscores the need for vigilant monitoring, as misattributing symptoms to unrelated causes can delay intervention and exacerbate complications.

Given the spectrum of presentations, from early febrile syndromes to severe systemic damage, clinicians must maintain a high index of suspicion when evaluating patients on hydroxyurea therapy [5–10]. Recognizing even mild initial symptoms may help prevent progression to severe complications. With advancing cancer treatments improving mortality, there is an even greater imperative to address treatment-related toxicity, morbidity, and mortality to optimize patient outcomes [12]. Timely discontinuation remains the cornerstone of management, preventing unnecessary morbidity and allowing safer alternative therapies, such as ruxolitinib, to be initiated for underlying myeloproliferative disorders [1, 2].  Table 1 summarizes previously reported hydroxyurea hypersensitivity cases along with the current case, highlighting the variability in onset, severity, and the necessity for early recognition to prevent life-threatening complications.

## 4. Conclusion

Hydroxyurea hypersensitivity, though rare, presents significant challenges in patient management. Early recognition and prompt discontinuation are essential to prevent severe complications, as delayed diagnosis can lead to life-threatening systemic inflammation. Hydroxyurea-induced fever is a clinically diagnosed hypersensitivity reaction, often confirmed through rechallenge, which consistently triggers fever recurrence. This pattern strongly supports a drug-related hypersensitivity rather than an infectious etiology. In most cases, symptoms resolve upon discontinuation, reinforcing the importance of timely intervention to prevent unnecessary morbidity.

Given its potential for severe inflammatory complications, careful monitoring is critical, particularly for patients who develop fever and flu-like symptoms following hydroxyurea initiation. Delayed hypersensitivity reactions, including pneumonitis, have been associated with significant morbidity and even fatal outcomes, underscoring the need for vigilance in recognizing atypical inflammatory responses.

Transitioning to alternative therapies such as ruxolitinib provides effective disease control without recurrence of hypersensitivity symptoms. Clinician awareness and patient education on recognizing drug-induced hypersensitivity reactions are vital to improving outcomes, safety, and overall treatment success.

## Figures and Tables

**Table 1:** Summary of hydroxyurea hypersensitivity cases.

Case	Symptoms	First onset	Confirmed on rechallenge	Management	Outcome
Lossos and Matzner [5]	Fever	1 day	Not reported	Discontinuation	Symptom resolution
van der Klooster et al. [6]	Fever	1-2 days	Yes	Discontinuation	Symptom resolution
Lannemyr and Kutti [7]	Fever	7 days	Yes	Discontinuation	Symptom resolution
Braester and Quitt [8]	Fever	21 days	Yes	Discontinuation	Symptom resolution
Sandhu et al. [9]	Severe pneumonitis, alveolar and interstitial inflammation, poorly formed granulomas	28 days	No	Discontinuation and systemic corticosteroids	Complete clinical and radiological resolution
	Severe pneumonitis, diffuse interstitial inflammation	3 months	No	Discontinuation and systemic corticosteroids	Fatal outcome despite treatment
Current case	Fever, chills, fatigue	28 days	Yes	Discontinuation, supportive care with acetaminophen and ibuprofen	Symptom resolution

## Data Availability

The data supporting the findings of this study are available from the corresponding author upon reasonable request.
